# Supplementary material for: Tuning electronic and phononic states with hidden order in disordered crystals
Source: Nat Commun. 2023 Jul 19;14:4328. doi: 10.1038/s41467-023-40063-w (PMC10356831; doi:10.1038/s41467-023-40063-w)
Supplement: Supplementary file 1 — Supplementary information [file 41467_2023_40063_MOESM1_ESM.pdf]

Supplementary Information

Tuning electronic and phononic states with hidden order in disordered crystals

Nikolaj Roth* & Andrew L. Goodwin

Supplementary Discussion 1 Powder Diffraction Patterns

Supplementary Figure 1 shows the calculated powder diffraction patterns for the three different disorder types in the 2D system. The upper part of the figure shows the full scale of the pattern. As these systems all have identical average crystal structures, the intensities of the Bragg diffraction peaks are identical. The difference in scattering comes in the diffuse signal, which is much weaker and not generally visible on the same scale as the Bragg peaks. In the lower sub-figure, the intensities have been multiplied by 100 to zoom in on the diffuse scattering, showing their differences.The noise seen in the diffuse scattering is due to a limited calculation size. Calculations were made by averaging over 100 configurations with each 60x60 atoms. To increase contrast of the diffuse scattering, Li was used as A atoms with O as B atoms. As shown in the main paper, single crystal scattering can be a better way to identify these phases.

Supplementary Discussion 2 Real space view of states

Fig. 4 a-d in the main paper shows a low-energy electronic mode between Γ and M composed of bonding s-orbitals from both atom types. In the ordered case the mode is delocalised with contributions from all s-orbitals, and the phase of the wavefunction changes slowly along the wavevector. For the randomly disordered system, this mode has now become localised. Atoms no longer contribute equally to the wavefunction, which consists of small coherent regions that are incoherent with respect to each other. For the two-in-two-out correlations, the mode is more delocalised, but not fully, and there is still incoherence in the phase. The mode becomes fully delocalised and coherent in the system with perpendicular strong bonds, and is similar to the ordered case. The same is not true for the mode shown in Fig. 4 e-h. This mode is on the flat band between M and X, consisting of the B atom p orbitals and A atom s orbitals. In the ordered system the band is localised on a single chain, and there is no mixing between chains. With random distortions there is mixing between directions but the mode is still localised. For this mode the two-in-two-out system is close to the random system, which was not the case for the first mode shown. However, for the system with perpendicular strong bonds, the mode is completely delocalised with strong coherence along chains in one dimension and mixing between chains along

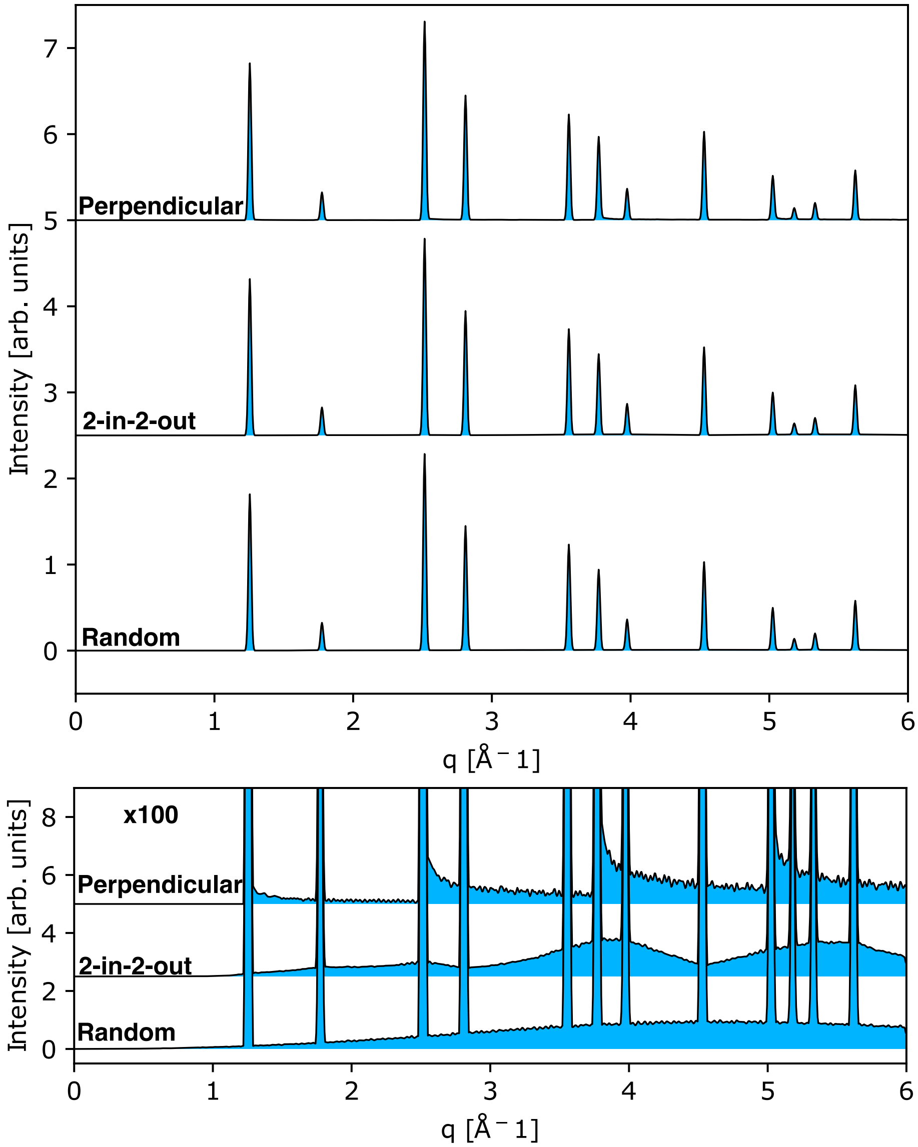

Supplementary Figure 1. Calculated Powder X-ray Diffraction patterns for the two-dimensional systems. Upper figure shows the full scale, while the lower figure shows the weak diffuse scattering signal by scaling by a factor of 100. The noise seen in the diffuse scattering is due to a limited calculation size.

the other dimension, causing chains to have phase shifts relative to each other. Thus, the electronic wavefunction can differ significantly depending on correlations, and in some cases the wavefunction can take on unique shapes not seen in ordered or randomly disordered systems.

Similar behaviour is seen for the phonon modes (Fig. 4 i-p). Here each atom is drawn as a circle and the colour now indicates the direction of movement while the saturation is again the amplitude. Arrows inside the circles highlight the displacement vectors further. Fig 4 i-l show a transverse acoustic shear mode between X and Γ. In the ordered system the mode is delocalised with layers three atoms wide moving out of phase with respect to each other, separated by a single layer of stationary A atoms. In the random system this mode becomes more localised with coherent regions that are incoherent with respect to each other. With the two-in-two-out correlations, the mode becomes more delocalised and consists of almost coherent layers moving as in the ordered case, but with disorder in the amplitude and directions of some atoms. In the system with perpendicular strong bonds, the mode becomes delocalised with layers moving like in the ordered case, but atoms between the layers are no longer stationary and move incoherently. This is in contrast to the optical mode shown in Fig. 4m-p, where the ordered system and the system with perpendicular strong bonds are both delocalised and coherent, while the random and two-in-two-out systems have incoherent localised modes.

Supplementary Discussion 3   Orbital contributions to states

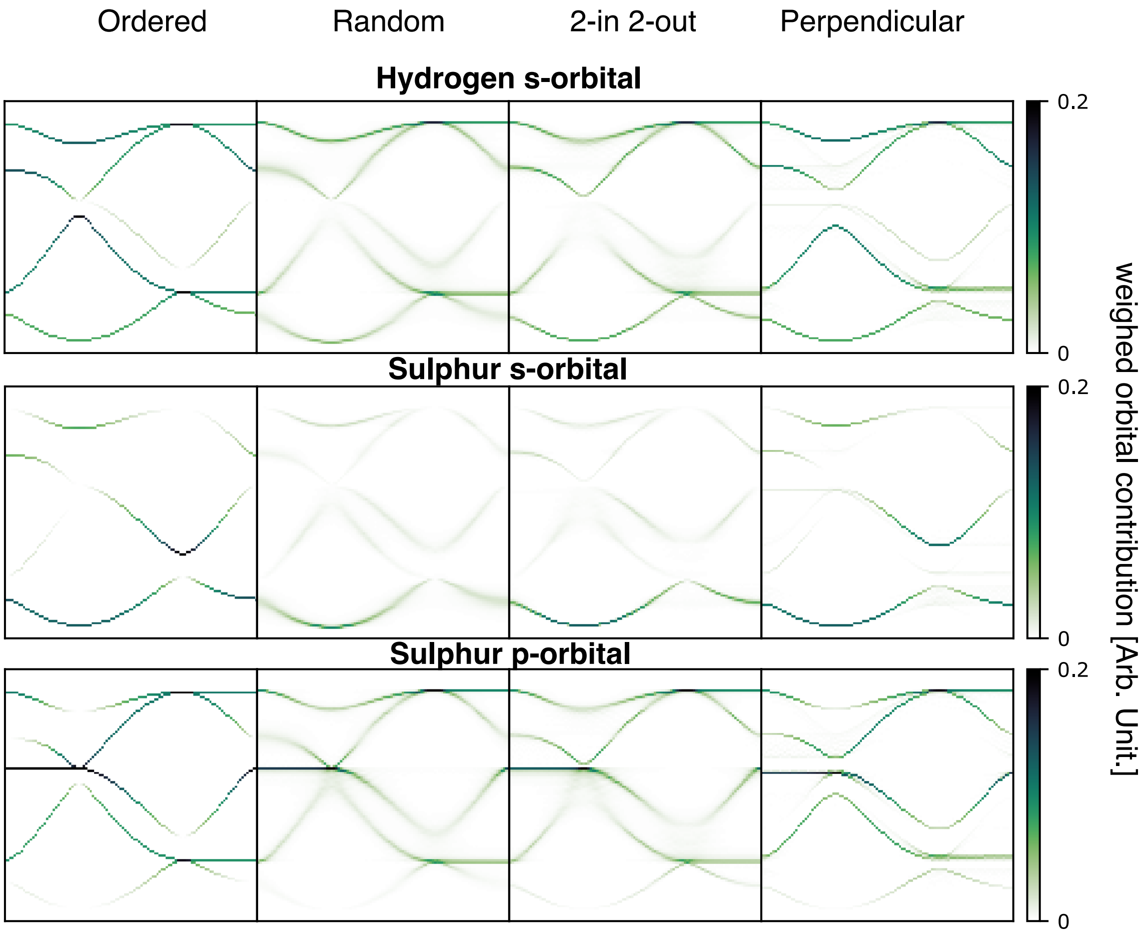

Supplementary Figure 2. Weighed orbital contributions to the electronic states in the 2D systems.

Supplementary Figure 2 shows the orbital contributions weighed by the number of modes in the 2D case.

Supplementary Discussion 4   Ordered versions

Supplementary Figure 3 shows the perpendicular hidden order state together with three examples of ordered versions obeying the same local chemical rules. For each of these the electronic and phononic bands are shown below. In general the band structures are quite similar, but the ordered versions have different weak bands which average to a broad set of states in the hidden order version. This is most clear around the M point phonon bands. The electronic and phononic density of states are shown in the bottom of the figure. The ordered versions have virtually identical density of states to the hidden ordered perpendicular system. The Fermi level also falls at the same position. Supplementary Figure 4 shows the total electronic energies for these systems. There is no driving force towards long range order as the ordered versions have the same total energy as the disordered version with the same local rules for bonding. Therefore the hidden order version would be favoured due to having higher entropy.

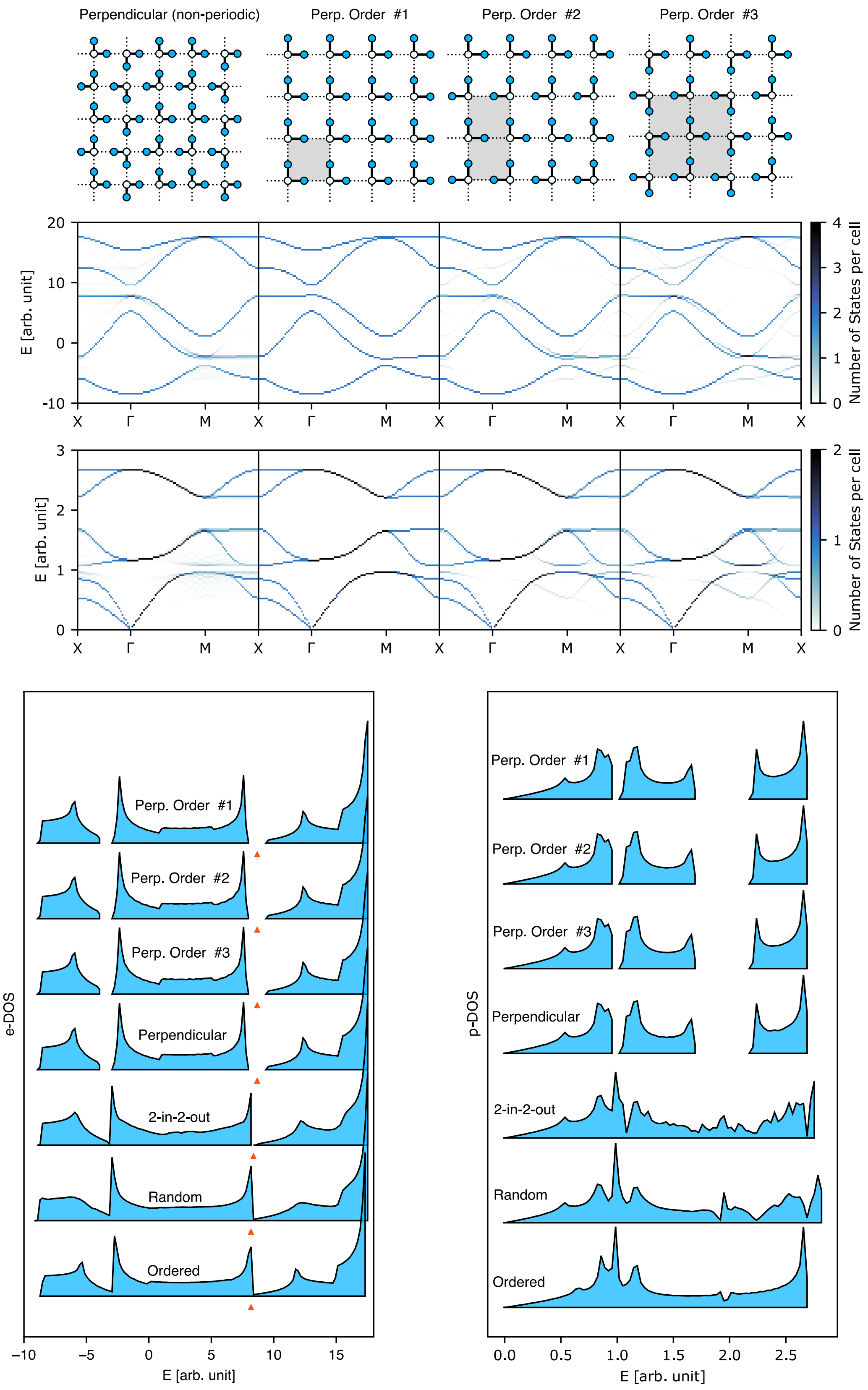

Supplementary Figure 3. Ordered versions of the 2D system with the same local rules as the 'perpendicular' hidden order system. Top row shows structures with the grey boxes indicating the unit cell of the periodic structures. Second and third row show the electronic and phononic band structures, respectively. Bottom left and right are the electronic and phononic density of states, respectively.

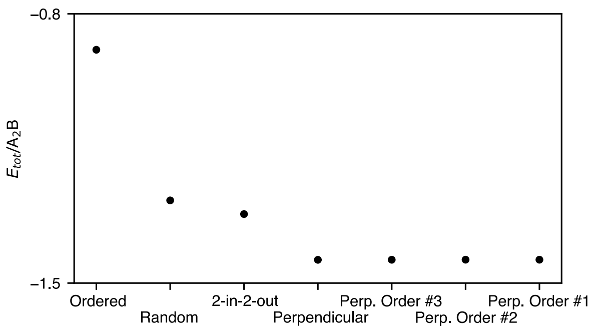

Supplementary Figure 4. Total electronic energy for ordered and disordered systems.

Supplementary Discussion 5 Three dimensional example

The same type of effects that were observed in 2D will also apply to three-dimensional systems. As an example, a 3D analogy to the 2D system is presented, based on a cubic A_3B structure with A atoms positioned between B atoms forming a simple cubic lattice. With A halfway between B sites, the system is ordered (Supplementary Figure 5a). If A atoms distort to form one strong and one weak bond to B sites, several disordered configurations can be obtained. With random distortions a wide variety of strong and weak bonds around B sites are generated (Supplementary Figure 5b). If instead each B site has the chemical rule of 3 strong and 3 weak bonds, a three-in three-out structure is obtained, as shown in Supplementary Figure 5c. In this structure there is a mixture of B sites with the 3 A atoms coordinated meridionally (mer) and facially (fac). Stronger chemical rules are then that the B atoms form only one of these, which in both cases leads to disordered systems shown in Supplementary Figure 5d and Supplementary Figure 5e for mer and fac geometry. Thus, in three dimensions more different types of short-range order can be generated due to the increased flexibility of another dimension.

Diffuse scattering patterns for these disordered systems are very different, while their Bragg diffraction intensities are identical. Supplementary Figure 5f-j shows the diffuse scattering down in the [111] plane of reciprocal space. While the ordered system has no diffuse scattering and the random disorder has broad smeared out diffuse scattering, the systems with local correlations have structured scattering. In the three-in three-out case pinch-points are again seen, reminiscent of other systems with similar rules [Fennell et al., Science 326, 415–417 (2009).]. In the meridional system the diffuse scattering has con-

densed further to form ring-like features with several maxima, while the facial system has strong narrow lines indicating long-range correlations of the disorder.

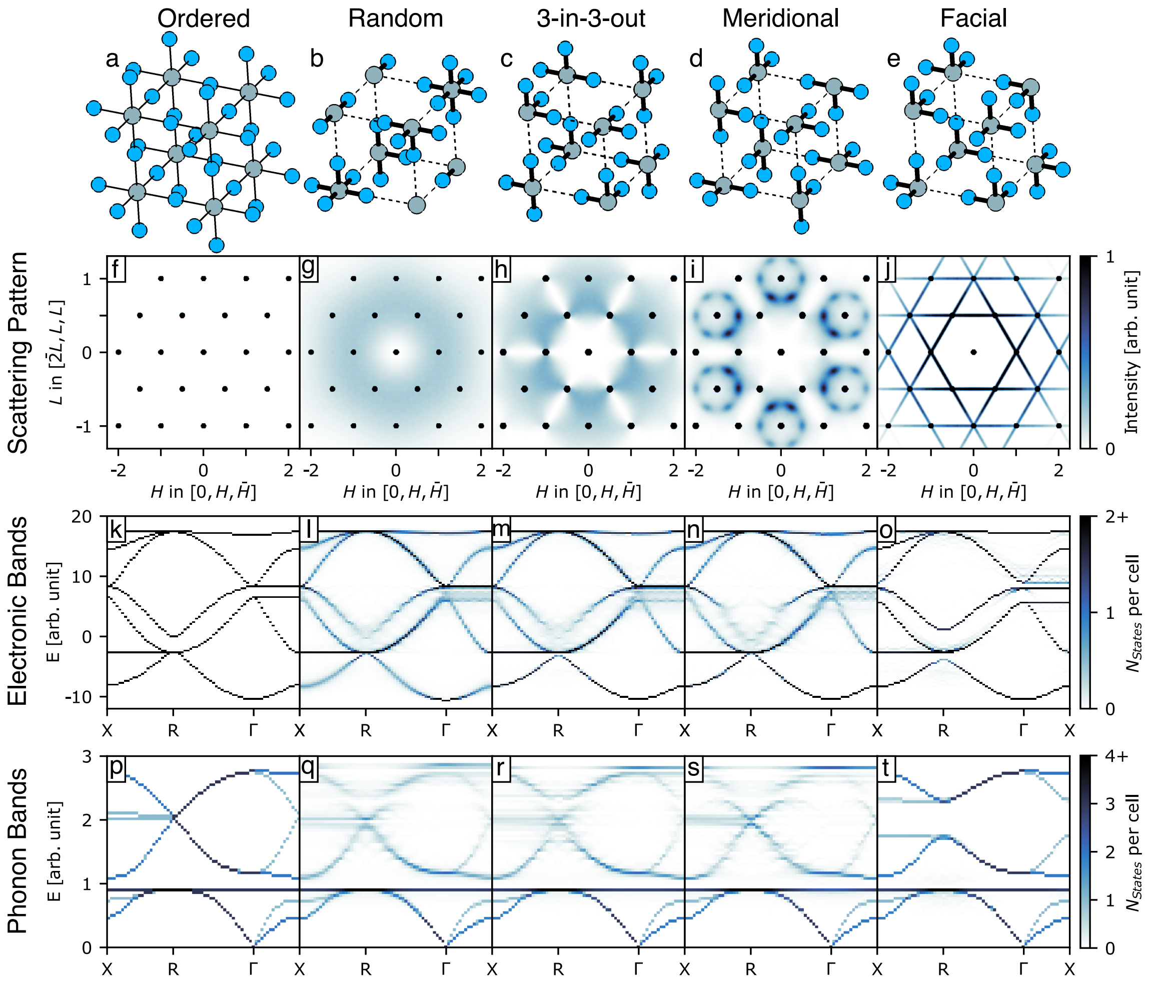

Supplementary Figure 5. Effects different disorder correlations on electronic and phonon bands. a) Ordered A_3B structure with A (blue) halfway between B (grey) on a simple cubic lattice. b) A random configuration of A site distortions. c) three-in three-out rule producing a mixture of meridional and facial geometry. d) meridional geometry only. e) facial geometry only. f-j) Corresponding diffuse scattering patterns. The hexagonal grid of black dots are Bragg peaks, which are several orders of magnitude stronger than diffuse scattering. k-o) Electronic bands for these systems. The energy scale is arbitrary and the zero point does not imply the fermi level. p-t) Phonon bands.

Similar trends to the two-dimensional system are found in the electronic (Supplementary Figure 5k-o) and phonon bands (Supplementary Figure 5p-t). Here the electronic bands are calculated using parameters for the R-3m phase of H_3S [Akashi, Phys. Rev. B 101, 075126 (2020)]. Phonon modes are calculated using parameters chosen to highlight effects of correlations. While random disorder tends to strongly broaden features that were present in the ordered system, correlated disorder changes the band dispersions and broadening, opening and closing band gaps. In the electronic bands (Supplementary Figure 5k-o) the low-energy band crossings at the R and Γ points can be opened to different sizes for the different types of correlations. The meridional system introduces a new band-like feature with a maximum at the R point in the electronic structure as well as new weak band-like features in the phonon modes. The facial system induces new band gaps for both electronic and phonon modes.
